# Continuous intraocular pressure monitoring in patients with obstructive sleep apnea syndrome using a contact lens sensor

**DOI:** 10.1371/journal.pone.0229856

**Published:** 2020-03-03

**Authors:** Elena Carnero, Jean Bragard, Elena Urrestarazu, Estefanía Rivas, Vicente Polo, José Manuel Larrosa, Vanesa Antón, Antonio Peláez, Javier Moreno-Montañés

**Affiliations:** 1 Department of Ophthalmology, Clínica Universidad de Navarra, University of Navarra, Pamplona, Navarra, Spain; 2 Department of Physics and Applied Mathematics, University of Navarra, Pamplona, Navarra, Spain; 3 Sleep Disorders Unit, Department of Neurophysiology, Clínica Universidad de Navarra, University of Navarra, Pamplona, Navarra, Spain; 4 Department of Ophthalmology, Hospital Miguel Servet, University of Zaragoza, Spain; Harper University Hospital, UNITED STATES

## Abstract

**Purpose:**

To analyse nocturnal intraocular pressure (IOP) fluctuations in patients with obstructive sleep apnea syndrome (OSAS) using a contact lens sensor (CLS) and to identify associations between the OSAS parameters determined by polysomnographic study (PSG) and IOP changes.

**Method:**

Prospective, observational study. Twenty participants suspected of having OSAS were recruited. During PSG study, IOP was monitored using a CLS placed in the eye of the patient. The patients were classified according to the apnea-hypopnea index (AHI) in two categories, severe (>30) or mild/moderate (<30) OSAS. We evaluated several parameters determined by the IOP curves, including nocturnal elevations (acrophase) and plateau times in acrophase (PTs) defined by mathematical and visual methods.

**Results:**

The IOP curves exhibited a nocturnal acrophase followed by PTs of varying extents at which the IOP remained higher than daytime measurement with small variations. We found significant differences in the length of the PTs in patients with severe OSAS compared to those with mild/moderate disease (P = 0.032/P = 0.028). We found a positive correlation between PTs and OSAS severity measured by the total number of apneic events (r = 0.681/0.751 P = 0.004/0.001) and AHI (r = 0.674/0.710, P = 0.004/0.002). Respiratory-related arousal and oxygen saturation also were associated significantly with the IOP PT length.

**Conclusions:**

Periods of nocturnal IOP elevation lasted longer in severe OSAS patients than those with mild/moderate OSAS and correlate with the severity of the disease. The length of the nocturnal PT is also associated to respiratory parameters altered in patients with OSAS.

## Introduction

Obstructive sleep apnea syndrome (OSAS) is a breathing disorder with a high prevalence. It is characterized by repeated episodes of upper airway collapse during sleep that cause sympathetic stimulation, hypertension, awakenings, hypoxia and hypercapnia in the patients. Chronically untreated OSAS is considered a risk factor for other diseases such as systemic hypertension, heart alterations, neurovascular diseases and type II diabetes [1–4]. An overnight sleep study polysomnography (PSG) is required for definitive diagnosis of OSAS. The disease severity can be classified based on the apnea-hypopnea index (AHI) as severe >30, moderate (15–30), mild (5–14.9)and normal <5[2].

Glaucoma is an optic neuropathy characterized by the progressive degeneration of retinal ganglion cells eventually causing blindness. Glaucoma affects around sixty million and is the second most common cause of loss of sight worldwide [5]. Approximately 2.4% of population older than 40 years have glaucoma; this percentage increases to 7% in those older than 70 years [5,6]. For this reason, the prevalence of glaucoma is projected to be higher with the population growth and ageing [7]. Several studies have established a relation between OSAS and glaucoma, the increased prevalence of glaucoma in patients with OSAS and the significant association between glaucoma and the OSAS severity have been reported [8–13]. Likewise, a higher prevalence of OSAS has been found in patients with glaucoma [14,15]. However, whether OSAS is a risk factor in glaucoma onset or progression is still under current debate.

Among the risk factors implicated in progression of glaucoma, the intraocular pressure (IOP) is currently the only controllable factor [16,17] and to reduce high IOP is the unique target of the available treatments [18,19]. Several groups have found a relation between IOP changes over long periods of time and deterioration of the visual fields (VFs) found in glaucoma patients [20–22]. IOP variations also occur during short time periods. Daily IOP values follow a circadian rhythm; several authors have reported a nocturnal increase of IOP values [23–27]. Some studies have indicated that this IOP elevation might be greater in patients with glaucoma compared to healthy controls [28,29]. Moreover changes in the bodily and/or head positions are associated with IOP variations; thus, the IOP increases with movement from standing and sitting to a supine position, and with neck movements outside the neutral position [30–32]. Additionally, ocular movements, blinking, or sleep phases can induce IOP fluctuations [33]. How these IOP fluctuations can contribute to the onset and progression of glaucoma is not completely understood. It is believed that the IOP increase could directly damage the optic nerve but also this elevation could affect the blood flow indirectly harming the optic nerve [34–36]. Finally, it has been described that astrocytes in the optic nerve head are mechanosensitive and can respond to the mechanical stress induced by IOP increase, reducing the trophic support or changes in extracellular remodeling in the optic nerve [37,38]. The association between an elevated IOP, as principal risk factor of glaucoma, and the presence of OSAS is still unclear.

A hindrance to establish the role of IOP fluctuations in the onset and/or progression of glaucoma has been the lack of a continuous IOP monitoring system. Clinicians obtain most IOP measurements during the day while the patient is sitting and the neck is in the neutral position, a practice that can underestimate the actual IOP. To ensure more reliable IOP studies, Sensimed AG (Lausanne, Switzerland) developed a contact lens sensor (CLS) that allows 24-hour semi-continuous monitoring to detect IOP variations and peaks throughout the day. The device is a soft silicon contact lens designed to stay on the corneal surface for up to 24 hours. The CLS has a micro-sensor that measures circumferential changes in the corneoscleral area. Leonardi et al. reported that these variations were correlated significantly with the IOP fluctuations[39]. The measurements or “bursts” are the median of these 30-second periods measured every 5 minutes, and they are considered a data point on the IOP graphs.

The purpose of the current study was to determine the changes in the IOP during sleep in patients who underwent PSG to diagnosis OSAS. We evaluated the correlation between the OSAS severity and several IOP-associated parameters in the study patients.

## Methods

### Participants

Twenty Caucasian participants (16 men, 4 women; mean age, 53.60 ± 10.35 years) were included in this prospective study. They were selected among patients subjected to polisomnographic study (PSG) in the Sleep Disorders Unit, at the Department of Neurophysiology, Clínica Universidad de Navarra. We selected patients with a high suspicion of OSAS based on their medical records, they received the details of the study and provided written informed consent for study inclusion. The presence of OSAS, determined by PSG, was confirmed in all patients except for patients 3 and 17 who had an apnea-hypopnea index <5. The patients were older than 18 years with non-ocular diseases except for ocular hypertension in the case of patients 7 and 14.

They were classified based on the AHI as having mild <15, moderate 15–30, or severe >30 OSAS. The exclusion criteria were previous corneal surgeries, corneal abnormalities (scars, leucomas ectasias), a previous diagnose of glaucoma, and severe dry eye (Schirmer test <5 mm). In order to discard the previous presence of glaucoma, we reviewed the medical histories of the patients, some of them followed in our department. Table 1 shows the anthropometric data of the patients and the prescribed medication during the study.

**Table 1:** Patient demographic data and medical records.

Patient	Age (yrs)	Gender	Weight (kg)	Height (cm)	Body mass index	Sistolic/ Diastolic Blood pressure (mmHg)	Diseases (Cause OSAS screening)	Medication	Initial IOP R/L
**1**	55	Male	97	178	30.6	130/84	Suspected OSAS	Omeprazole/Diclofenac	10/12
**2**	46	Male	84	186	24.3	114/74	Nasal Septum Deviation	None	14/13
**3**	46	Female	62	170	21.3	100/63	Insomnia /Headache	Trazodone/Lormetazepam	15/14
**4**	40	Male	102	186	29.5	110/70	Headache	Ibuprofen PRN	14/18
**5**	67	Male	96	183	28.7	140/86	Hypertensive Cardiopathy	Lisinopril/ Lercanidipine/ Doxazosin/Esomeprazole	17/17
**6**	64	Male	91	173	26.9	146/86	Headache	Omeprazole/Losartan/Bisoprolol/ Acetylsalicylic acid/ Rosuvastatin/ Escitalopram	15/17
**7**	47	Male	98	Unkn	Unkn	Unkn	Suspected OSAS	Brimonidine/Timolol (ocular)	22/21
**8**	53	Male	84	184	24.8	145/94	Headache	Ibuprofen PRN	20/17
**9**	42	Male	102	184	30.2	135/100	Arterial Hypertension	Olmesartan/Amlodipine /Hydrochlorothiazide	21/20
**10**	44	Female	89	162	33.9	100/70	Asthenia	Hydrocortisone/Fludrocortisone/ Desmopressin/ Acetylsalicylic acid/ Atorvastatin/ Pantoprazole	12/13
**11**	51	Male	96	176	31.0	125/75	Asthenia	Pravastatin/Iron supplement, Pantoprazole, Diclofenac	18/15
**12**	65	Male	80	172	27.0	119/81	Headache	None	18/14
**14**	41	Male	Unkn	Unkn	Unkn	Unkn	Suspected OSAS	Latanoprost (ocular)	17/17
**15**	56	Female	99	170	34.3	120/70	Arterial Hypertension	Enalapril/Atorvastatin	17/18
**16**	56	Male	90	166	32.7	170/100	Nasal Septum Deviation	Enalapril	14/14
**17**	37	Female	Unkn	Unkn	Unkn	106/71	Insomnia /Headache	Duloxetine/ Tramadol/Acetaminophen/Pantoprazole/Amitriptyline/Iron/ Diazepam	9/9
**18**	73	Male	Unkn	Unkn	Unkn	100/65	Headache	Unkn	13/16
**19**	61	Male	82	164	30.4	Unkn	Headache	Metformin, Tepazepam	12/13
**20**	50	Male	114	190	31.6	110/70	Arterial Hypertension/Snoring	Candesartan	8/7
**21**	65	Male	78	165	28.7	121/73	Arterial Hypertension	Perindopril/ Indapamide	15/13

We measured the initial IOP in both eyes by pneumotonometry (Table 1) and placed the CLS in the eye of the patients between 4 and 6 pm the evening before the start of PSG. The right eye was chosen to place the CLS in all patients except patient 11 who was placed in the left eye. The following day between 9 and 10 am the CLS was removed from the eye using conjunctival forceps under topical anesthesia and the data obtained were analyzed. During wake periods, the patients received artificial tears to minimize discomfort. The ethics committee of Navarra approved the study protocol, which was conducted in accordance with the Declaration of Helsinki.

### Instrumentation

**Fig 1:**
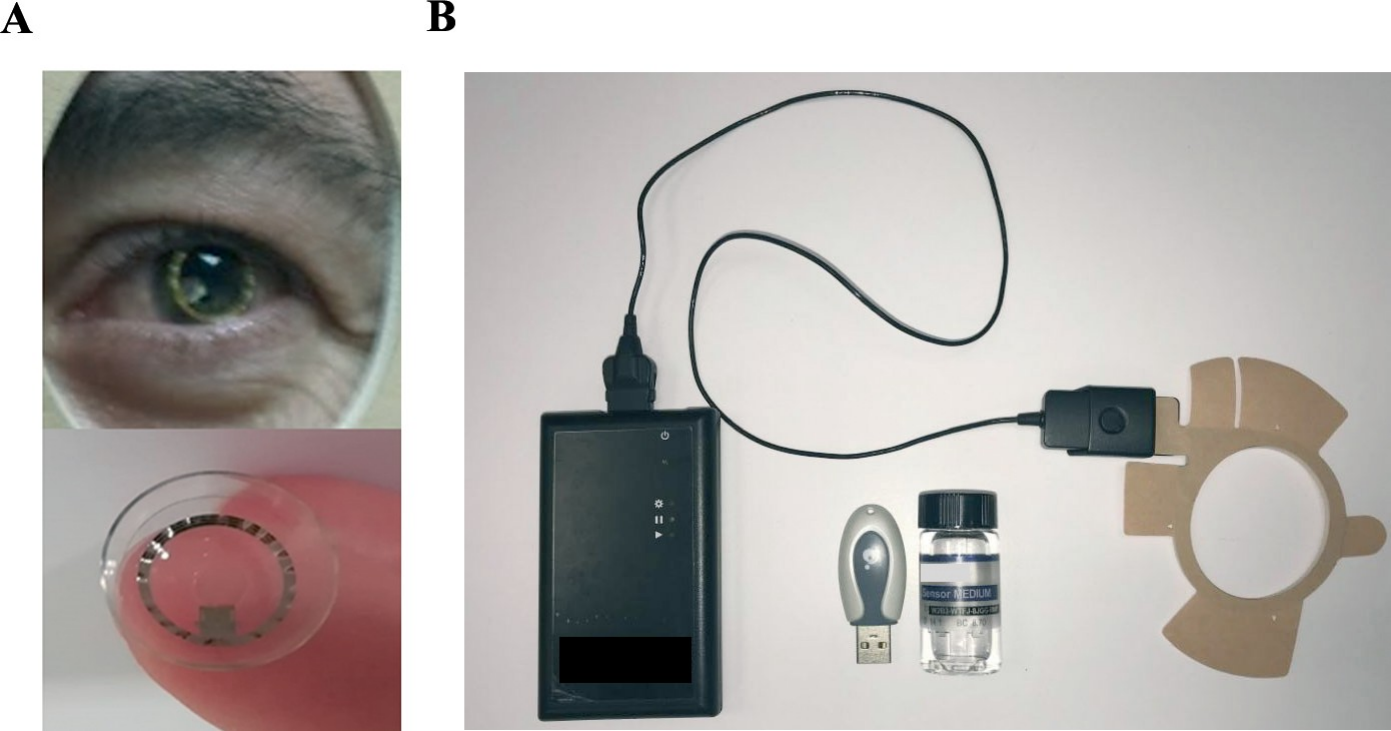
Sensimed Triggerfish system. A. Sensimed Triggerfish is a soft silicon contact lens sensor (CLS) that contains two strain gauges able to detect changes in the corneal shape. The lens has embedded a microprocessor and the antenna. B. CLS transmits strain gauge data to an adhesive antenna that sends information to a recorder. The CLS selected based on the curvature of the cornea is placed in the eye of the patient. The adhesive antenna is attached to the area around the eye and connected through a cable to the portable recorder that is carried by the patient.

The Sensimed Triggerfish contact lens (Sensimed AG) detects changes in the ocular circumference and corneal curvature at the corneoscleral junction that is associated with IOP fluctuations (Fig 1).

The changes in ocular volume are detected by a strain gauge. After being placed in the eye of a patient, the CLS measures 300 continuous events for 30 seconds every 5 minutes that correspond to a “burst”. The values obtained were received by an antenna placed around the patient eye and transmitted to a portable recorder by a cable. The acquired data stored in the recorder was transferred to the computer for analysis. We obtained the raw data containing every value detected by the CLS and the median value for every set of measures (burst) that we used in the study to create the IOP curves.

### Polysomnographic study (PSG)

All patients underwent baseline PSG to confirm the clinical suspicion of OSAS. Nocturnal sleep was recorded with Harmonie 5.2 (Stellate, Montreal, Canada) using Lamont 32-Sleep amplifiers (Lamont Medical, Wisconsin, USA) as the regular sleep studies in our Unit. Frequency of digitization was 200 Hz. The recording included EEG, tibial and chin electromyography, electrooculography, oronasal flow, respiratory effort, oximetry, heart rate, and body position. An electrocardiogram was recorded with two derivations (V3 and V5) that were amplified band-pass filtered (0.30–30 Hz) and digitized at 500 Hz. The sleep stage classification was performed according to the current American Academy of Sleep Medicine (AASM) criteria[40]. The AASM score criteria also were used to establish the hypopneic and apneic events and arousals. The parameters obtained from PSG are described in Table 2.

**Table 2:** Patient polysomnographic data.

Patient	AHI	Maximum apnea duration (sec)	Average apnea duration (sec)	Non-respiratory arousals index	Arousal respiratory index	Oxygen saturation < 90%	Average oxygen saturation %	Total sleep time (min)	Sleep latency (min)	Sleep efficiency %	Waking period %	% N1 (Total sleep time)	%N2 (Total sleep time)	%N3 (Total sleep time)	%REM (Total sleep time)	Number of nocturnal awakenings
1	61.15	118.3	41.0	15.38	36.94	38.4	91	340	17	77	18.04	2.65	68.92	24.01	4.42	6
2	37.47	102.1	36.8	5.57	3.24	0	97	388	7	93	2.87	1.94	39.61	38.97	19.48	3
3	2.54	31.9	22.1	13.29	0	0	97	307	67	54	32.89	2.44	31.43	46.09	20.03	6
4	47.34	110.4	29.4	2.55	30.16	4.0	94	472	36	88	3.81	2.01	55.57	19.83	22.59	9
5	57.19	112.2	41.5	0	64.58	4.3	96	374	19	78	15.90	2.41	37.75	39.36	20.48	19
6	16.600	53.1	21.5	33.21	15.47	0	96	477	12	81	17.05	6.29	46.75	22.96	24.00	13
7	53.79	70.5	17.1	15.60	48.04	2.7	94	585	4.5	92	6.82	2.22	58.94	22.50	16.34	11
8	28.14	44.8	19.2	24.03	21.50	0.4	96	380	33.5	70	20.59	1.45	58.10	24.11	16.34	7
9	15.42	39.2	17.0	11.30	6.11	0	97	404	29.5	87	5.40	2.97	49.07	30.11	17.84	11
10	8.63	36.9	20.1	4.40	3.55	1.2	93	355	9.5	75	5.73	7.76	27.22	42.45	22.57	4
11	61.13	85.1	21.1	1.88	33.70	3.2	94	380	19	85	12.42	2.61	49.06	23.49	24.84	3
12	36.40	68.6	24.0	3.40	26.19	0	96	406	23.5	69	30.21	2.59	45.75	23.06	28.61	8
14	21.45	55.7	22.5	4.26	0.61	0	98	414	77.5	76	13.53	4.59	43.36	36.47	15.58	13
15	22.98	87.2	21.2	4.24	18.88	0.2	93	410	9	73	33.05	5.00	40.85	30.85	23.29	13
16	74.06	96.1	22.0	4.17	65.27	2.0	95	519	6.5	90	7.91	2.31	40.79	40.79	16.10	10
17	4.52	26.4	15.2	1.98	1.13	0	96	425	145.5	66	18.24	5.76	51.18	18.00	25.06	14
18	31.91	105.5	34.1	0.92.	29.67	0	96	263	17.5	72	32.75	23.85	52.42	10.88	12.86	23
19	13.23	84.0	24.5	5.58	12.75	1.9	94	377	26	80	13.81	3.32	45.29	28.15	23.24	7
20	65.41	113.4	30.4	2.06	62.70	2.7	95	466	NA	74	35.62	1.72	65.99	17.60	14.70	4
21	33.25	65.8	26.4	0.94	15.34	5.8	95	513	6.5	80	23.51	9.76	61.17	11.12	17.95	17

### Sensimed Triggerfish data analysis

We analyzed the IOP fluctuations during the apneic-hypoapneic events, normal breathing periods during sleep as well as awakening times in patient 1–12. To do that, we determined the beginning and end of each event using the raw data and the median of every period was obtained.

We evaluated several parameters associated with the IOP graphs obtained during PSG. We calculated the wake/sleep slope, determined by linear regression based on the IOP measurements obtained 1 hour before sleep and those obtained 1 hour after sleep onset; in the same manner, we calculated the sleep/wake slope with the IOP values 1 hour before and 1 hour after the awakening. We also determined the extent of the IOP acrophase defined by the measurement of time in which the IOP increases, starting when the patient rests in bed and ends when the IOP reaches the maximum elevation. The IOP acrophase was followed by a variable plateau time (PT) during which the IOP showed small variations.

**Fig 2:**
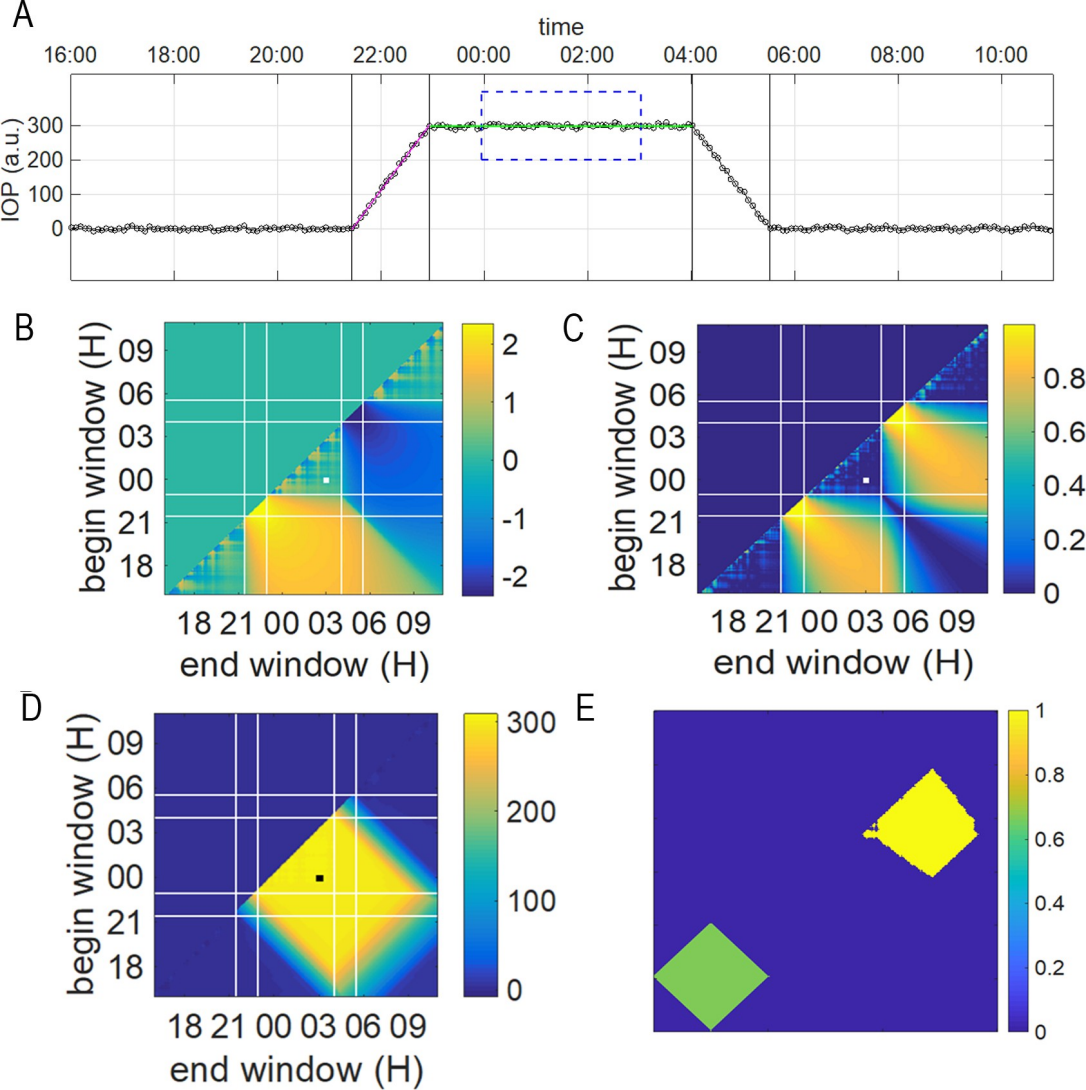
Method for the determination of the duration of the IOP plateau B. The time series of the IOP (insert A) is transformed into two-dimensional plots (inserts B-D). In insert A, an example window beginning at time 00:00 and ending at time 03:00 is drawn with a blue dashed line. The data enclosed in the window are used for evaluating inserts B-D. In B, the slope of the linear regression of the window’s data is reported as a color code plot (in log scale). In C, the corresponding R^2 coefficient is reported. In insert D, the median value corresponding to the window is reported. In order to construct inserts B-D all beginning, and end times are considered for defining the covering windows. In insert D, the plateau localization is extracted as a square pattern with a 45-degree orientation with respect to the axes. The values corresponding to the example window shown in insert A is reported by small marked squares in insert B-D for illustrative purpose. In insert 2E the approximate yellow square upper right corner is a binarized picture of Fig 1D obtained by fixing a threshold value. The threshold value is associated with the data distribution percentile. The case shown here corresponds to the 80^th^ percentile. The rotated green square (template) is chosen to have the same area as the yellow square and is translated around to seek for the optimum cross-correlation between the template and the binarized pictured. An associated norm (between 0 and 1) gives the level of correlation (here for the 80^th^ percentile, we get a correlation of 0.891) and the best location for the superposition of the template and signal.

The plateau was measured according to two strategies. Using the first strategy (plateau A), three doctors examined the graphs to reach a consensus on a PT duration. The second strategy (plateau B) followed a signal processing approach based on measuring the median value on moving windows of varying widths and use of Matlab software (Matlab and Statistics Toolbox Release 2017b, The MathWorks, Inc., Natick, MA) to automatically detect the PT of the IOP. The algorithm is first explained and illustrated below on synthetic data and thereafter used for the 20 study patients. The algorithmic sensibility and its statistical justification will be published elsewhere (Bragard et al. Automatic detection of plateaus in ocular physiological data, manuscript in preparation). We provide a summary of the technique here. A variable width and moving window covers part of the signal. The information of the signal covered by a specific window is reported in two-dimensional graphs (Fig 2A–2D).

The two axes of these graphs correspond to the beginning time (Y-axis) and the end time (X-axis) of the corresponding covering window, respectively. This technique is inspired and has been previously described in a study of neuromotor control[41]. From the data enclosed in the windows (see caption of Fig 2), we report the slope of a linear interpolation shown in Fig 2B and the coefficient of determination (R^2) for the corresponding linear interpolation (displayed in Fig 2C). We also report the median value of the data enclosed in the window as shown in Fig 2D. We worked directly on Fig 2D to identify the plateau time (PT) and Fig 2B and 2C were used for the acrophase determination. From the observation of Fig 2D we identify the presence of a plateau with a square rotated by 45 degrees with respect to the (X-Y) axes. The basis and difficulty of the method lies in the setting of a threshold value to determine the boundary of the square in Fig 2D. The threshold value transforms Fig 2D into a binary figure (black and white, 0 and 1 only) as shown in the upper right part of Fig 2E.

**Fig 3:**
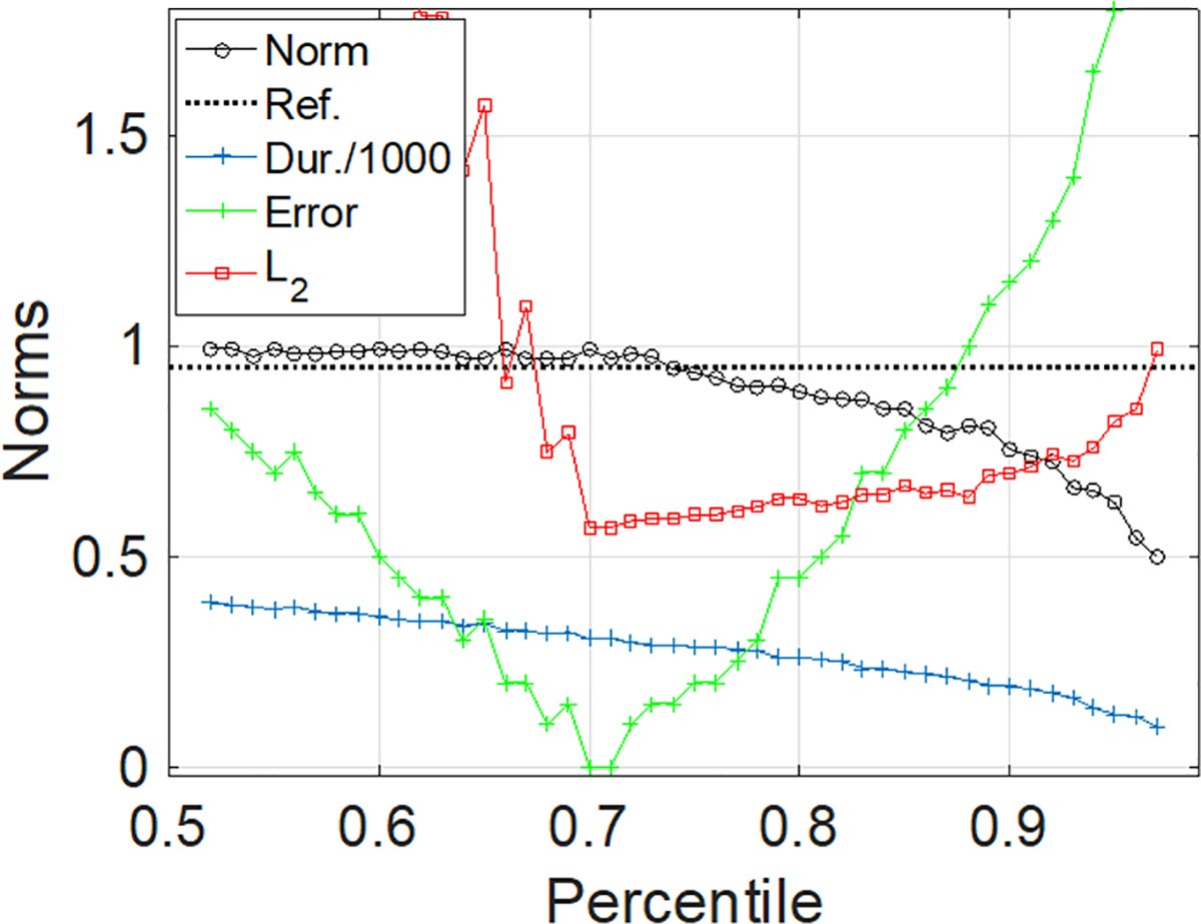
Different indicators used for the determination of the PT B. The circle symbols indicate the optimum cross-correlation between template and signal at different values of the percentile (cut-off values). The dotted line indicates the 0.95 level for reference purpose. The blue curve represents the corresponding PT duration (in minutes and rescaled by 0.001 to fit in the vertical scale) extracted from our method at the different percentiles. The green curve represents the error of the PT determination (see text Material and Methods for definition). The error is vanishing for percentiles 70th and 71st indicating that the method gives a perfect PT determination for such percentile values. The red curve (with square symbols) is a L2 norm (see text Material and Methods for definition) that is expected to be minimum for the percentile corresponding to the plateau detection.

The next step of the method consists in searching for the best match of the resulting binarized object with an ideal template, i.e., a square with the same area (see Fig 2E, lower left corner) that is rotated by 45 degrees with respect to the axes. The built-in function that searches for the best match optimization is directly available in the Matlab software and is called with the function name “normxcorr2”. At this stage, because the choice for the threshold value for the binarization of the pattern is arbitrary, we scan all possible percentiles from the data as possible percentiles. We report the best correlation between the binary object and the square template pattern as a function of the percentile values as shown in Fig 3 (indicated by the black circles).

For this illustrative synthetic case we know where the plateau starts and ends and therefore we can evaluate the error as the sum of the difference in absolute values between the estimated and real time of the beginning and end of the plateau (shown in Fig 3 by the green curve). However, for all the patients considered here we need to calculate the beginning and end of the plateau, therefore we need a measure that is comparable to the error. Here we have chosen as a measure of the error the norm L_2_ defined as follows:

$$L_{2}=\frac{1}{Np}\sqrt{\sum_{i=1}^{Np}{\left(y_{i}-median(y)\right)}^{2}}$$

where Np is the number of points in the detected plateau, and median(y) is the median value for the detected plateau. Indeed, we observe that the norm L_2_ is minimum when the plateau is detected. As a further check we ensure that when the plateau is detected, the correlation given by “normxcorr2” is above the 95% cross-correlation that is taken as an additional cut-off value. The plateau duration provided by the two strategies, plateaus A and B, are compared in the Results section.

### Statistical analysis

The Shapiro-Wilk test was used to determine if the samples deviated significantly from normal distribution. Thus, differences between the severe and non-severe groups were analyzed by the Student t-test for parametric data; the Mann-Whitney U-test was applied to the non-parametric parameters. To find associations between the parametric variables, we determined Pearson’s correlation coefficient for normal distributions or Spearman’s correlation coefficient in non-parametric variables. The statistical study was performed using SPSS software version 20.0.1 (SPSS Inc., Chicago, IL).

## Results

### Subjects

Twenty patients who met the inclusion criteria underwent PSG (Table 1). Among the 20 patients, eleven had severe OSAS, five moderate OSAS, and four mild or no OSAS according to the OSAS classification (Table 1). We divided the patients in two groups based on the apnea-hypopnea index, severe group (AHI>15) and non-severe group (AHI<15). There were no significant differences between groups in age, height, weight, body mass index, or blood pressure (*P* > 0.05) (Table 3). We also reviewed the medical histories and concomitant medication use of the patients (Table 1).

**Table 3:** Comparison of physical parameters between severe and non-severe patient groups.

	Non-severe	Severe	
	Mean ± SD	Mean ± SD	*P* value
**Age**	49.3 ± 9.5	55.9 ± 10.3	0.16
**Weight**	87 ± 13.3	93.5 ± 10.9	0.284
**Height**	172.5 ± 8.7	178 ± 9	0.239
**Systolic Blood Pressure**	121.7 ± 20.4	123.9 ± 19.7	0.828
**Diastolic Blood Pressure**	79.1 ± 14.1	77.8 ± 10.2	0.822
**Body Mass Index**	28.8 ± 4.8	29.3 ± 2.6	0.785

p value

* p<0.05

** p ≤ 0.01.

The CLS was well tolerated generally; only patient 18 decided to have the sensor removed before the end of the study. For this reason, we excluded this patient for the IOP study. Fourteen patients had intense or mild conjunctival hyperemia that resolved in less than 24 hours.

### Study of continuous IOP in patients with OSAS

IOP pressure was measured before sensor placement for each patient (Table 1). IOP values were considered in a normal range in all patients. Only patient 7 showed an IOP of 22 mmHg, slightly elevated. The patient had been previously diagnosed with high IOP and treated for this reason with topical medication.

**Fig 4:**
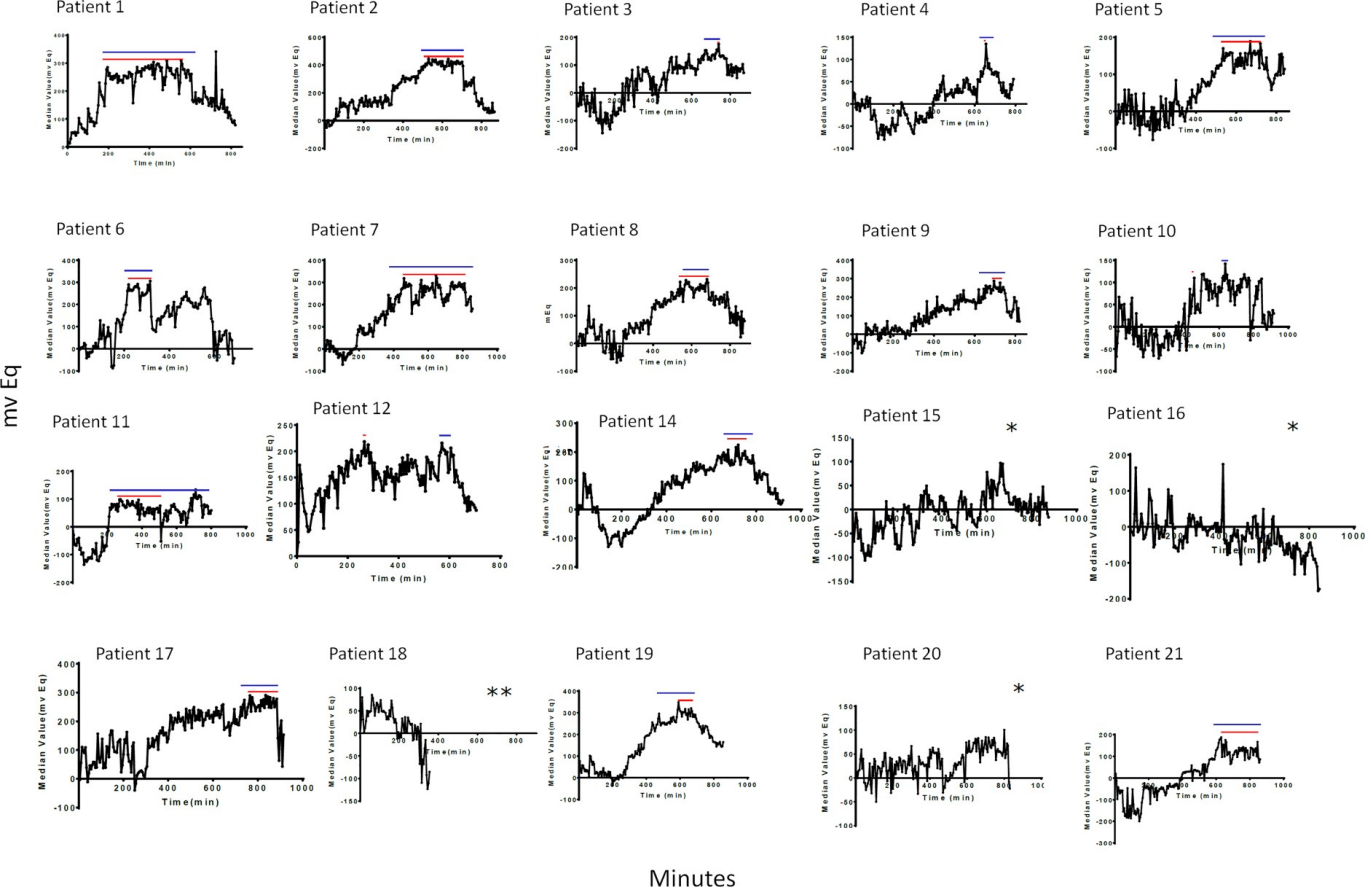
Spectrograms of signal profiles obtained from the CLS Sensimed Triggerfish in the 20 study eyes. Patient 18 opted to remove the CLS before the end of the PSG. Red lines show the length of plateau A and the blue lines display the extent of plateau B. * Patients 15,16 and 20 do not show a IOP plateau. **Patient 18 opted to remove the CLS before the end of the PSG.

The analysis of the IOP fluctuations obtained by the sensor showed significant nocturnal IOP increases (IOP acrophase), while patients were in bed compared to daytime, which confirmed previous observations [23,24]. (Fig 4).

Patients 15, 16, and 20 did not have IOPs with significant nocturnal acrophase. These patients had great fluctuations in the measurements, while the median IOP values remained similar during the study (Fig 4). We measured the IOP acrophase when the patient was lying in bed and the wake-to-sleep and sleep-to-wake slopes (Table 4).

**Table 4:** Evaluation of curve parameters obtained by the CLS during PSG.

Patient	OSAS Severity	Acrophase (min)	Wake to Sleep Slope	Sleep to wake Slope	Plateau A (min)	Plateau B (min)
**1**	**Severe**	65	1.51	-1.12	345	410
**2**	**Severe**	195	1.61	-1.9	170	185
**3**	**Non- Severe**	115	0.35	-0.73	0	125
**4**	**Severe**	120	0.03	-0.44	0	110
**5**	**Severe**	185	0.21	n.d.	180	240
**6**	**Non- Severe**	152	1.11	-2.09	100	120
**7**	**Severe**	260	1.08	-1.09	354	425
**8**	**Non- Severe**	225	1.02	-0.57	140	115
**9**	**Non -Severe**	504	0.92	-1.36	30	90
**10**	**Non -Severe**	125	0.67	-0.35	0	45
**11**	**Severe**	55	1.94	-0.54	225	570
**12**	**Severe**	205	0.62	-0.85	15	105
**14**	**Non-Severe**	340	1.07	0.71	100	170
**15**	**Non -Severe**	n.d.	n.d.	n.d.	n.d.	n.d.
**16**	**Severe**	n.d.	n.d.	n.d.	n.d.	n.d.
**17**	**Non-Severe**	565	0.71	-1.07	110	125
**18**	**Severe**	n.d.	n.d.	n.d.	n.d.	n.d.
**19**	**Non-Severe**	335	1.24	-0.88	86	250
**20**	**Severe**	n.d.	n.d.	n.d.	n.d.	n.d.
**21**	**Severe**	430	0.07	-0.14	230	300

After the acrophase, the IOP peaks remained fairly constant for a varying extent that differ depending on each patient and we refer to it as the plateau time (PT). We determined the PT in every patient except patients 15, 16 and 20 (Table 4). In most patients, it remained stable until the patient awakened and arose from bed (Fig 4). We followed two different approaches, the first, PT A, was direct visual examination of the plateau magnitude by three different observers. The second, PT B, was obtained through the previously described mathematical approach (Table 4). Both methods showed a high level of correlation (r = 0.821, *P*<0.001) (Table 5).

**Table 5:** Analysis of the correlation between the IOP curve obtained values and the PSG parameters.

		Arousal respiratory events	Non-respiratory arousals index	Time saturation lower of 90%	Total number apnea-hypopnea	Apnea-Hypopnea Index	Plateau A	Plateau B	Wake to sleep slope	Sleep to Wake Slope	% N1 Sleep Phase	% N2 Sleep Phase	% N3 Sleep Phase	% REM Phase
**PlateauA**	***r***	0.548*	0.120	0.499*	0.681**	0.674**		0.821**	0.426	-0.119	-0.069	0.626**	-0.353	-0.589*
	**p**	0.028	0.658	0.049	0.004	0.004		<0.001	0.100	0.673	0.798	0.009	0.180	0.016
**PlateauB**	***r***	0.540*	-0.116	0.538*	0.751**	0.710**	0.821**		0.506*	0.045	-0.154	0.460	-0.304	-0.310
	**p**	0.031	0.669	0.032	0.001	0.002	<0.001		0.046	0.873	0.570	0.073	0.253	0.243

*r* correlation coefficient, p p value

* p<0.05

** p ≤ 0.01. Spearman/Pearson correlation test

### Relation of IOP and OSAS severity

We evaluated the correlation between the apneic episodes and IOP fluctuations in the first 12 patients with different degrees of OSAS. We did not obtain significant differences in the IOP comparing non apnea vs apnea episodes. Moreover, we did not find specific IOP fluctuations or peaks associated with the apneic episodes compared to post-apneic normal sleep breathing or waking hours.

**Fig 5:**
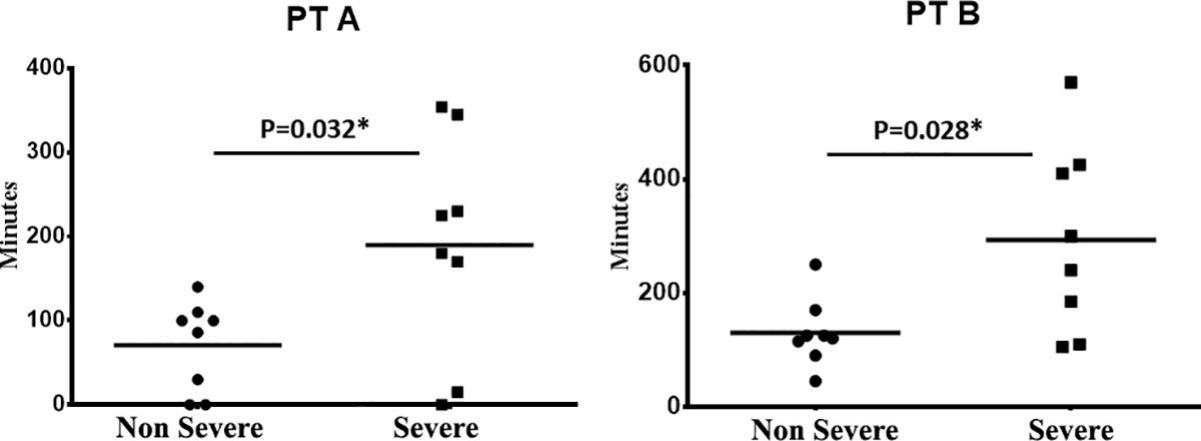
Comparison of measured plateaus in patients with Severe or Non-Severe OSAS. The duration of the Plateaus obtained by the two methods A and B was significantly longer in the severe OSAS patients compare to those with a mild/moderate disease.

Then, we focused on the curve created by the CLS signal and analyzed different parameters, wake-to-sleep slope, acrophase duration, PT, and sleep-to-wake slope. We found significant differences in PT when we categorized the patients as having severe and non-severe disease. The comparison shows the longer plateaus times in the patients with severe disease compared to those with non-severe disease (*P* = *0*.*032* and *P = 0*.*028*) (Fig 5).

PT B showed a positive association with the wake to sleep slope (r = 0.506, *P* = 0.046) (Table 5).

We also evaluated associations between the parameters obtained with the PSG data that defined OSAS degree (Table 2) and the values we have determined in the IOP curve (Table 4). The PT A and PT B correlated positively with the OSAS severity determined by the AHI and the total number of apneic events (Table 5). Moreover, we found a positive relation between the plateaus and the number of arousals due to respiratory events (r = 0.548/ 0.540, *P* = 0.028/0.031) (Table 5). The plateau time is negatively associated with the oxygen saturation meaning that patients who have better oxygen saturation show shorter plateaus. In this sense, if we analyze the amount of time that patients remain with a saturation below 90%, this is associated positively with the plateau length (r = 0.499/0.538, *P* = 0.049/0.032) (Table 5).

The PT A was correlated positively with the N2 sleep phase (r = 0.626, *P* = 0.009) and negatively with the REM sleep periods (r = -0.589, *P* = 0.016).

We did not find any association between the initial IOP measured before the study and the parameters of the curve. Moreover, there were not statistical difference between the initial IOP in severe and non-severe patients (*P* = 0.652)

In summary, we found that PTs during sleep were associated with apneic severity in OSAS patients. The results suggested that an increased number of arousals related to respiratory causes and a lower oxygen saturation also are related to a longer PT.

## Discussion

We used a contact lens sensor (CLS) to determine IOP changes during sleep, including pre and post sleep periods. The IOP changes have been determined by mvEq units measured every 5 minutes since the CLS was placed in the eye until it was removed. The data collected using this system resembled a real situation, because the IOP was measured while the patient remained asleep and undisturbed. The CLS was considered safe in the current study and the patients tolerated the CLS well, with only one patient discontinuing the study and deciding to remove the lens referring a foreign-body sensation. Fourteen patients had transient conjunctival hyperemia that resolved 24 hours after lens removal. These adverse events occurred frequently but were mild and they have been reported previously [42–44].

We measured the IOP fluctuations once for each study patient. Repeated measurements are always desirable to establish the system reliability; however, several studies have already reported very good reproducibility with the Sensimed CLS for IOP measurements, for example, Mansouri et al. reported the high duplicability of the system by performing two measurements with the CLS in the same patients 6 to 8 days apart[45]. Due to the nature of the current study that was associated with PSG we obtained only one recording per patient.

Several groups have tried to answer questions related to accuracy and validity of the CLS IOP values by comparing the data obtained from the CLS with traditional IOP measurements obtained either from the fellow eye or from the same study eye before or after placement of the CLS. Mansouri et al. also reported a highly significant correlation between both measurements using the IOP in the fellow eye for comparison,[42] and Aptel et al. also reported this association[46]. Other studies, however, have reported a lower correlation between both values [47,48].

As we described previously, literature have established an association between the presence of OSAS and glaucoma, however, only a few studies have attempted to evaluate the IOP fluctuations in patients with OSAS. Thus, Huseyinoglu et al. found small differences in the IOP depending on the OSAS severity; however, the authors failed to find a correlation between the AHI and IOP [49]. The literature has provided contradictory results, with some studies reporting a positive correlation between the AHI and IOP,[11,50–52] while others did not [53]. Each of these studies obtained a few IOP measurements during the ocular examination. The first study that evaluated continuously the IOP in patients with OSAS and used SENSIMED triggerfish as a IOP sensor was done by Shinmei and collaborators. They have examined the impact of apnea-hypopnea events and IOP values in seven patients, reporting a significant decrease in the IOP associated to apnea-hypopnea events in four out of seven patients [54]. Interestingly these fluctuations have no impact in the global IOP curves [54], showed a similar pattern to those obtained in our study (Fig 5).

We performed a similar analysis evaluating the IOP fluctuations during the apneic episodes versus non apneic periods in the first 12 patients with different degrees of OSAS. We did not find significant differences in the IOP comparing non apnea vs apnea episodes. We have focused on the analysis of the IOP curves in the OSAS patients, most of the curves showed a pattern. We observed an IOP elevation starting when the individual lies on the bed. The IOP reaches a plateau (PT) that usually finish when the patient wakes up and leave the bed with a decrease in the IOP that creates a negative slope. We found that patients with severe OSAS had significantly longer PTs compared to those with moderate or mild OSAS. We also found an association between the PT duration and the apneic severity evaluated by the AHI and the total number of apneic and hypopneic events (Table 5). We observed that patients with severe OSAS tends to reach the plateau in the early hours during bedtime and the IOP was maintained at that high level during longer periods. In this sense, we found a statistically significant association between the slope of the acrophase and the duration of the plateau (Table 5). Being high IOP a risk factor for glaucoma disease, severe OSAS could contribute among other factors to glaucoma onset and be more prevalent in these patients.

Several studies using the CLS have established the presence of a nocturnal acrophase in a great number of individuals; some studies have suggested that this acrophase is more frequent in patients with glaucoma (80% to 90%) than healthy subjects (70%)[29]. Results also have suggested that enlargement of this peak happens more frequently in patients with glaucoma compared to healthy subjects [29]. The nocturnal acrophase and subsequent plateau could contribute to disease onset and progression.

The PT also was correlated with other respiratory parameters related to OSAS such as the arousal related to the respiratory events or the duration of severe hypoxic conditions. Recurrent hypoxia besides vascular alterations described before that might affect the optic nerve, activates the renin-angiotensin system (RAS)[55], causing an increase in the choroidal blood flow that can ultimate lead to an increase in elevated episcleral venues pressure [56]. The ocular RAS has been also implicated in formation of aqueous humor, the drainage of which modulates IOP [57–60]. Therefore, several researchers have shown that RAS inhibition with angiotensin-converting enzyme (ACE) inhibitors or angiotensin II type I receptors blockers is beneficial in both normotensive and glaucomatous eyes [61–63]. Moreover, the IOP has decreased in patients treated orally with ACE inhibitors (captopril) or AT-1 receptor blockers (losartan) [64,65]. These data suggested that RAS activation could partly explain the IOP increase in patients with OSAS.

Patients 15, 16, and 20 had greater IOP fluctuations over time and not an overall change in contrast to the nocturnal acrophase widely described in the other patients (Fig 5). Analysis of the specific characteristics of these patients showed the commonalities: the age range and the presence of arterial hypertension treated with an antihypertensive drug in monotherapy. Two patients were treated with enalapril, an ACE inhibitor, and the third with candesartan, an AT1 receptor antagonist. The treatment might have been related to the “abnormal” IOP pattern observed by the mechanism described above. Others also were treated with these drugs but in combination with other antihypertensive agents. These patients had at least partially the nocturnal acrophase and plateaus (Fig 5). We cannot explain these phenomena; further studies should confirm these preliminary observations that associate these treatments and IOP fluctuations.

The current study had some limitations, with the first related to the population characteristics and the small sample size. The number was small compared to other studies using the same system; the reason could be that patients with OSAS are less aware of the risk of uncontrolled high IOP than patients with glaucoma, more responsive to wearing a CLS. Another limitation was due to the CLS properties; IOP variations were measured in mvEq, which cannot be converted to mmHg, the conventional pressure unit. Extrapolating the obtained data to conventional IOP values is a limitation. A contact lens can modify the corneal shape and thickness, which might have affected the values obtained. In this sense some authors have pointing out that the measures obtained by the CLS after several hours can be affected by these changes losing accuracy[66]. Finally, the CLS signal can be noisy and these large fluctuations should be analyzed in a subsequent study; in the current study the fluctuations due to bodily positions were minimized due to PSG. Most patients were supine during the monitoring except for patient 6 who alternated between supine and lateral positioning. Despite these limitations, this study can contribute to understand better la relationship between OSAS and glaucoma.

In conclusion, the Sensimed Triggerfish allowed evaluation of the IOP fluctuations in patients with different OSAS severities. The results indicated that patients with OSAS might have normal IOP measurements during medical visits but their IOPs might be elevated substantially at night, the phenomenon has been also described for the general population. In this study we have observed that IOP increase remains elevated, creating a plateau, which lengths are significantly longer in patients with severe OSAS based on the AHI and total number of apneic/hypopneic events. We also found an association between high IOP and low oxygen saturation, both parameters that have been related to glaucoma progression.

Further prospective studies are needed to demonstrate that the length of the nocturnal IOP elevations in patients with OSAS has a role in glaucoma onset and/or progression.
